# Effects of resveratrol on cell growth and prolactin synthesis in GH3 cells

**DOI:** 10.3892/etm.2014.1544

**Published:** 2014-02-13

**Authors:** WANG CHAO, ZHANG XUEXIN, SU JUN, CHU MING, JIN HUA, GUOFU LI, CHUNLEI TAN, WANHAI XU

**Affiliations:** 1 Department of Neurosurgery, The Fourth Affiliated Hospital of Harbin Medical University, Harbin, Heilongjiang 150001, P.R. China; 2 Department of Neurosurgery, The Third Affiliated Hospital of Harbin Medical University, Harbin, Heilongjiang 150001, P.R. China; 3 Department of Neurosurgery, The First Affiliated Hospital of Harbin Medical University, Harbin, Heilongjiang 150001, P.R. China; 4 Department of Urology, The Fourth Affiliated Hospital of Harbin Medical University, Harbin, Heilongjiang 150001, P.R. China

**Keywords:** resveratrol, estrogen, prolactinoma, apoptosis

## Abstract

Resveratrol (RE), a phytoestrogen, has antiestrogenic properties. Estrogen plays a key role in the development and progression of pituitary prolactinoma. Moreover, RE is a potent cancer chemopreventive agent that inhibits the initiation, promotion and progression of carcinogenesis. The present study investigated the antitumor effects of RE on GH3 pituitary tumor cells. A concentration- and treatment duration-dependent biphasic effect of RE on the proliferation of the GH3 cells was demonstrated. After three days of treatment, RE stimulated proliferation at low concentrations and inhibited proliferation at high concentrations. However, when the treatment duration was reduced to 6 h, RE inhibited proliferation in a concentration-dependent manner. In addition, RE induced apoptosis with the activation of caspase-3 and -8, and decreased the percentage of prolactin (PRL)-immunopositive GH3 cells. Furthermore, RE suppressed expression of the PRL gene and inhibited the cell proliferation and PRL synthesis induced by 17β-estradiol (E2). In GH3 cells, the proliferation response exhibited higher sensitivity to E2 compared with the PRL response; by contrast, the PRL response was more sensitive to RE than the proliferation response was. These results indicate that RE, an antiestrogenic compound, exerts its antitumor effect on GH3 cells through the suppression of GH3 cell growth and through the inhibition of PRL synthesis. The RE-induced cell apoptosis was shown to be caspase-dependent. Therefore, the present study provides support for the use of RE in the chemoprevention and chemotherapy of pituitary prolactinoma.

## Introduction

Pituitary prolactinoma is an estrogen-related tumor (1). Estrogen regulates the synthesis and secretion of prolactin (PRL) from lactotrophs (2). Numerous studies have demonstrated that estrogen induces lactotroph proliferation and results in prolactinoma formation (3,4). The effects of estrogen are mediated by estrogen receptors (ERs) (5), which are expressed in all pituitary tumor subtypes (6). Furthermore, ER expression levels are higher in prolactinoma than in other pituitary tumor types and may be correlated with tumor size (6,7). Therefore, close correlations exist among estrogen, ERs and prolactinoma, which provide a viable therapeutic target in prolactinoma treatment. Certain antiestrogenic compounds have been shown to exert a suppressive effect on the cell growth and PRL secretion of normal pituitary cells and pituitary tumor cells (8).

Resveratrol (RE), a type of phytoestrogen, acts as an agonist as well as an antagonist for ERs (9). In certain tumors, RE has shown chemopreventive and chemotherapeutic effects; this may be attributed to its capacity to inhibit cellular events associated with the initiation, promotion and progression of tumors (10). These properties indicate the potential use of RE as an adjuvant in the chemotherapy of prolactinoma.

GH3 cells, an established estrogen-responsive cell line from rat pituitary tumor cells, secrete PRL and growth hormone (GH) (11). These cells are a useful model for investigating the effects of RE on prolactinoma. In the present study, the effect of RE on the synthesis of PRL and cell proliferation in GH3 cells was examined.

## Materials and methods

### Reagents and chemicals

The following reagents and chemicals were used in the present study: RE (Sigma, St. Louis, MO, USA); 17β-estradiol (E2; Sigma); general caspase inhibitor z-VAD-fmk (Promega Corporation, Madison, WI, USA); and antibodies against rat PRL (rPRL; Santa Cruz Biotechnology, Inc., Santa Cruz, CA, USA), GH (Chemicon, Temecula, CA, USA), poly ADP ribose polymerase (PARP; Invitrogen Life Technologies, Carlsbad, CA, USA) and caspase-3 and -8 (Santa Cruz Biotechnology, Inc.).

### Cell culture

GH3 cells were obtained from the Institute of Basic Medical Sciences of the Chinese Academy of Medical Sciences (Beijing, China) and were maintained in Ham’s F-10 medium (Gibco-BRL, Carlsbad, CA, USA) containing 12.5% horse serum (Gibco-BRL), 2.5% HyClone^™^ fetal bovine serum (ThermoFisher Scientific, Waltham, MA, USA), 2 mmol/l L-glutamine (Sigma), 0.25 μg/ml Fungizone^®^ (Invitrogen Life Technologies) and 80 μg/ml gentamicin (Sigma). The cells were plated at various densities and were incubated for four days in the maintenance medium. Prior to treatment, the medium was replaced with the treatment medium, a defined serum-free, phenol red-free medium, containing Ham’s F-12 medium (Gibco-BRL) with 10 μg/ml insulin (Sigma), 5 μg/ml transferrin (Sigma) and 0.5 ng/ml parathyroid hormone (Sigma). After 24 h, cells were treated with or without E2 and RE at various concentrations for different time periods.

### Cell proliferation assays

Cell proliferation was assessed via an MTT assay (Sigma) following extensive validation, which included a direct comparison to tritiated thymidine incorporation and a linear correlation with the actual cell number. Briefly, 1×10^5^ GH3 cells were plated in 96-well plates. Following treatment, with or without the addition of E2 and RE at various concentrations for 6 h or three days, 20 μl MTT at a concentration of 5 mg/ml was added to each well. After 4 h, 200 μl dimethyl sulfoxide was added and the optical density at 490 nm was observed using a Fluostar Optima ABS UV/Vis microplate reader (BioTek Instruments, Inc., Winooski, VT, USA). Data were presented as a percentage of vehicle-treated control values.

### Apoptosis analysis

GH3 cells were treated with vehicle control or RE, or RE in the presence or absence of pretreatment with 100 μM Z-VAD-fmk, a pan-inhibitor of caspase for 1 h. Following treatment, apoptosis was assessed using the Annexin V-fluorescein isothiocyanate (FITC)-labeled apoptosis detection kit I (BD Biosciences Pharmingen, San Diego, CA, USA). Cells were washed twice with phosphate-buffered saline (PBS), suspended in binding buffer and stained with Annexin V-FITC and propidium iodide. Cells undergoing apoptosis were detected by flow cytometry FACS Calibur (Becton Dickinson, Rutherford, NJ, USA).

### Immunofluorescent microscopy

GH3 cells were cultured on glass coverslips in a 24-well plate. Dual-labeling immunofluorescent detection of GH and PRL was performed using primary antisera obtained from two different species of animal. The cells were initially incubated in monkey antiserum against rat GH (1:500) in a humidified chamber for 1 h at room temperature and rinsed with PBS three times, followed by incubation in rabbit antiserum against rPRL (1:1,000). The immunoreacted coverslips were washed three times with PBS, incubated with tetramethylrhodamine isothiocyanate-labeled goat anti-monkey IgG (Sigma), followed by incubation in FITC-labeled chicken anti-rabbit IgG (Sigma) in a humidified chamber for 30 min at room temperature. Immunofluorescence (GH, red; PRL, green) was observed with a laser scanning confocal fluorescence microscope LSM 710 (Carl Zeiss AG, Oberkochen, Germany). The fields were systematically scanned across the coverslip to avoid overlap. Each group consisted of three coverslips and >1,000 cells were observed per coverslip. The proportions of each cell type were determined.

### Reverse transcription-polymerase chain reaction (RT-PCR) of PRL

Total RNA was prepared from GH3 cells using TRIzol LS reagent (Invitrogen Life Technologies) according to the manufacturer’s instructions. Messenger RNA was reverse transcribed into single-stranded complementary DNA (cDNA) using 1.0 mg total RNA, an oligo (dT) primer (Promega Corporation) and Moloney murine leukemia virus reverse transcriptase (Gibco-BRL). The reaction mixtures were diluted 20-fold and subjected to PCR amplification of PRL cDNA, as previously described (12). The PCR primers were designed on the basis of published sequences of rat PRL (13). Amplification of cDNAs was conducted using the following primers: Forward: 5′-CCT GAA GAC AAG GAA CAA GCC-3′ and reverse: 5′-TGG GAA TCC CTG CGC AGG CA-3′. PCR amplification was conducted using the GeneAmp^®^ PCR System 2400 (Perkin-Elmer, Norwalk, CT, USA) following denaturation of the samples for 5 min. Each cycle consisted of: Denaturation at 94°C for 1 min, annealing at 60°C for 1 min and extension at 72°C for 2 min. Following amplification, there was a final 10 min extension step at 72°C. qPCR analysis of PRL was conducted over 30 cycles. The PCR products were separated by electrophoresis on a 1.0% agarose gel, visualized with ethidium bromide staining and quantified via scanning densitometry using NIH Image software, version 1.61 (National Institutes of Health, Bethesda, MD, USA). The quantity of PCR products for PRL was normalized to that of the PCR products of glyceraldehyde-3-phosphate dehydrogenase per sample.

### Western blot analysis

Following treatment, cells were rinsed twice with ice-cold PBS solution. Lysis buffer (containing 50 mM Tris, pH 7.5; 5 mM ethylene glycol tetraacetic acid, 120 mM NaCl, 20 mM α-glycerophosphate, 1% NP-40, 15 mM sodium pyrophosphate, 50 mM sodium fluoride, 10 mM sodium orthovanadate, 0.5 mM phenylmethanesulfonyl fluoride, 10 μg/ml aprotinin, 10 μg/ml leupeptin and 20% glycerol) was added (120–180 μl) and the cells were incubated for 10–30 min at 4°C. Following cell scraping, cell lysates were clarified by centrifugation (9,600 × g for 15 min at 4°C) and the proteins in the supernatant were determined via a bicinchoninic acid protein assay (Pierce Chemical Co., Rockford, IL, USA). Equal quantities of proteins were used for western blot analysis. Briefly, 35 μg cell lysate was subjected to electrophoresis on 12% sodium dodecyl sulfate-polyacrylamide gel electrophoresis gel and the separated proteins were transferred onto polyvinylidene fluoride membranes. The membranes were washed twice with PBS and Tween-20 (PBST) and incubated with a blocking buffer (4% non-fat milk) for 1–2 h at room temperature. The membranes were incubated overnight at 4°C with primary antibodies (anti-PRL, anti-PARP, anti-caspase-3, anti-caspase-8 and anti-actin 1:5,000), followed by three 10-min washes with PBST. The secondary antibody was goat anti-rabbit IgG conjugated with horseradish peroxidase (Amersham Life Sciences, Buckinghamshire, England). Membranes were incubated with the secondary antibodies at room temperature for 1 h. Densitometric assays were conducted using a Kodak Digital Science ID scanner (Eastman Kodak, Rochester, NY, USA). This experiment was repeated three times.

### Statistical analysis

Each experiment was conducted three times, independently. Data are expressed as the mean ± SD and were analyzed using SPSS 10.0 software (SPSS Inc., Chicago, IL, USA). Statistical significance was determined using analysis of variance, Student’s t-test and a χ^2^ test. P<0.01 was considered to indicate a statistically significant difference.

## Results

### Biphasic effect of RE on the proliferation of GH3 cells

**Figure 1:**
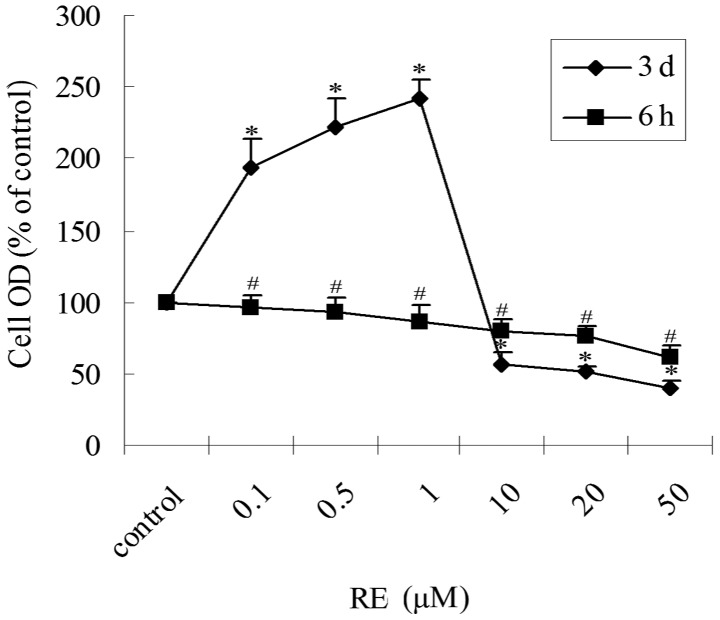
Effect of RE on GH3 cell proliferation. GH3 cells were treated with varying concentrations of RE for two different time periods and cell proliferation was assessed by an MTT assay. Data were calculated as a percentage of the control and expressed as the mean ± SD of three separate experiments. ^#^Indicates significant differences from the 6 h control values and ^*^indicates significant differences from the three day control values (P<0.01). OD, optical density; RE, resveratrol.

To determine the proliferation-related effects of RE, GH3 cells were treated with increasing concentrations of RE in serum-free, phenol red-free medium for 6 h or three days and cell proliferation was assessed via an MTT assay. In the cells treated with RE for 6 h, an inhibitory effect on cell proliferation was observed in a concentration-dependent manner (Fig. 1). However, treatment of GH3 cells with RE for three days had a biphasic effect on cell proliferation (Fig. 1). RE treatment for three days at low concentrations (0.1–1 μM) stimulated cell proliferation (2 to 3-fold) and at high concentrations (10–50 μM) inhibited proliferation (~2-fold) compared with the control. The half maximal inhibitory concentration (IC_50_), of RE was between 10 and 20 μM. Thus, depending on the concentration and duration of treatment, RE exhibited inhibitory and stimulatory effects on the proliferation of GH3 cells.

### RE-induced cell apoptosis is dependent on the activation of caspase-3 and -8 in GH3 cells

**Figure 2:**
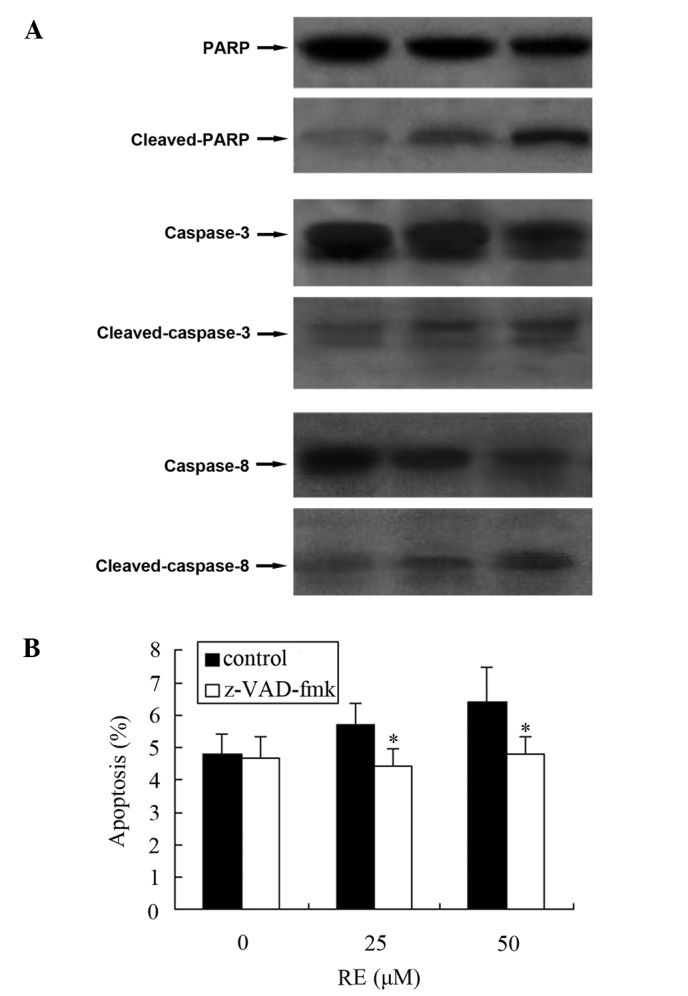
RE induced apoptosis through a caspase-dependent pathway. (A) GH3 cells were treated with 25 or 50 μM RE for 24 h and the expression of cleaved caspase-3 and -8 and PARP was detected by western blotting. (B) Cells were pretreated with 100 μM Z-VAD-fmk, a pan-inhibitor of caspase for 1 h, followed by treatment with 25 or 50 μM RE for an additional 24 h to determine the extent of apoptosis. PARP, poly ADP ribose polymerase: RE, resveratrol. Data are expressed as mean ± SD. ^*^P<0.05, compared with the vehicle group.

We have previously demonstrated that RE induces apoptosis in GH3 cells (14). As cells undergoing apoptosis execute programmed cell death by the activation of caspases and the cleavage of PARP, the levels of cleaved PARP, caspase-3 and -8 were measured. As Fig. 2A demonstrates, cleaved PARP was detected in GH3 cells, accompanied by cleaved caspase-3 and -8. To further confirm the involvement of caspase activation in the RE-induced apoptosis, a broad spectrum caspase inhibitor, z-VAD-fmk, was employed. After 24 h of RE exposure, the percentage of apoptotic cells was reduced by z-VAD-fmk (Fig. 2B).

### Effect of RE on the proportions of various cell types in GH3 cells

**Figure 3:**
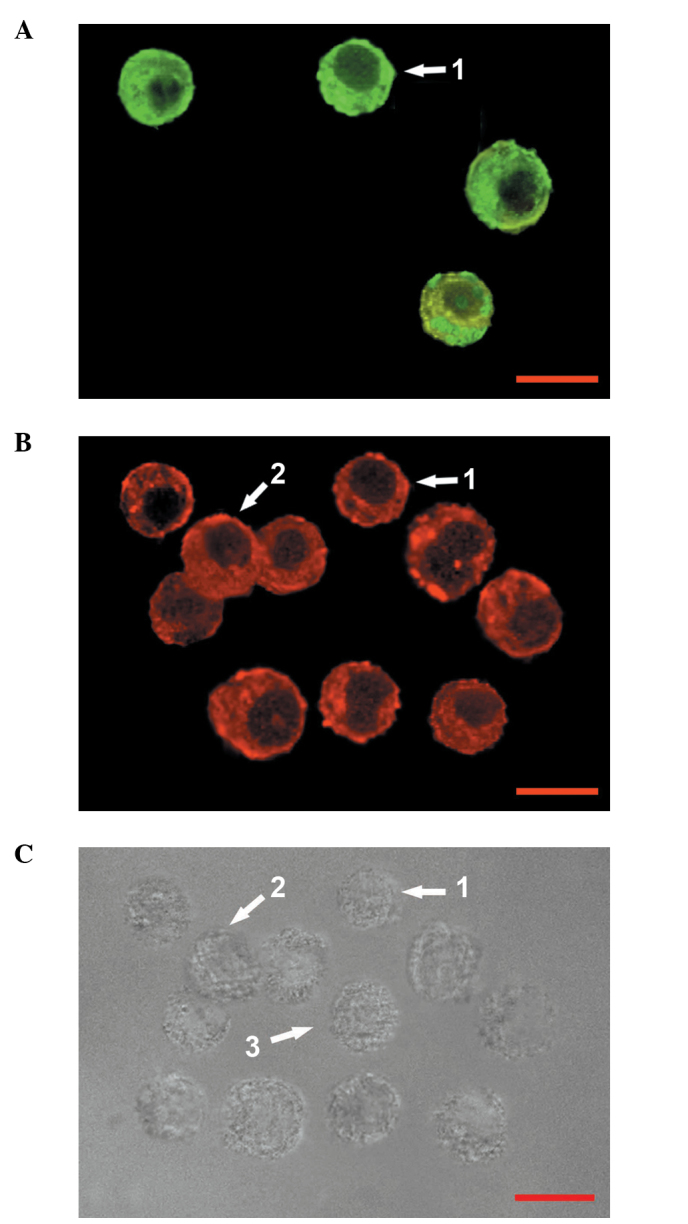
Double-immunostaining for PRL and GH in GH3 cells. GH3 cells were cultured in serum-free, phenol red-free medium for three days (control). GH3 cells were fixed and stained green (PRL) or red (GH) following reaction with anti-rat PRL and anti-rat GH antibodies. The images show the same field of a culture plate. (A and B) Immunofluorescence images showing PRL^+^ and GH^+^ cells, respectively. (C) Bright-field image obtained from confocal microscopy. Scale bar, 20 μm. 1; PRL^+^/GH^+^ cell, 2; GH^+^ cell, 3; PRL^−^/GH^−^ cell. PRL, prolactin; GH, growth hormone. Magnification, ×400.

GH3 cells may be divided into four categories: Cells immunoreactive for GH alone (GH^+^ cells); cells immunoreactive for PRL alone (PRL^+^ cells); cells immunoreactive for PRL and GH (PRL^+^/GH^+^ cells) and cells in which neither PRL nor GH were detected (PRL^−^/GH^−^ cells) (11). In the present study, double-labeling immunocytochemical analysis of GH3 cells cultured in serum-free, phenol red-free medium (control) for three days revealed that the GH3 cells were composed of PRL^+^/GH^+^ cells, GH^+^ cells and PRL^−^/GH^−^ cells (Fig. 3); PRL^+^ cells were not detected. At high concentrations (10–50 μM), under the same culture conditions, RE treatment for three days decreased the proportion of PRL^+^/GH^+^ cells; however, the proportion of GH^+^ cells increased. At low concentrations (0.1–1 μM), RE did not change the relative proportions of the types of GH3 cells (Table I) and PRL^+^ cells were not detected. The relative proportion of PRL^−^/GH^−^ cells was not affected by RE at the selected concentrations (0.1–50 μM). Although quantitative analysis of the immunoreactivity was not conducted at high concentrations of RE (10–50 μM), PRL immunoreactivity was observed to be decreased compared with that in the controls.

### Inhibitory effect of RE on PRL synthesis

**Figure 4:**
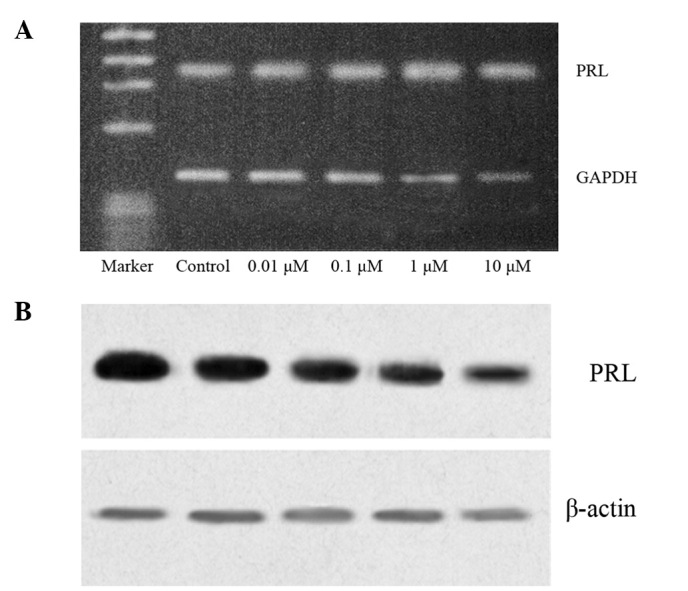
Dose-response experiment of RE inhibition for the expression of PRL. (A) GH3 cells were treated with RE (0.01–10 μM) for three days, total RNA was prepared and RT-PCR was conducted using specific primers for PLR and GAPDH. PCR products were resolved on a 1.0% agarose gel and visualized via ethidium bromide staining. (B) PRL concentration in the cell lysate was determined by western blot analysis and equal loading was confirmed by determining the actin content. Each band was quantified using a densitometer. PRL, prolactin; GAPDH, glyceraldehyde 3-phosphate dehydrogenase; RE, resveratrol; GH, growth hormone; RT-PCR, reverse transcription polymerase chain reaction.

The effect of RE on PRL at the mRNA level was evaluated using RT-PCR and the content of intracellular PRL was established by western blot analysis. GH3 cells, cultured in serum-free, phenol red-free medium (control), synthesized PRL. At selected concentrations (0.01–10 μM), RE reduced PRL mRNA and intracellular PRL levels, resulting in decreased PRL production (Fig. 4).

### Inhibitory effect of RE on cell proliferation and PRL synthesis induced by E2

**Figure 5:**
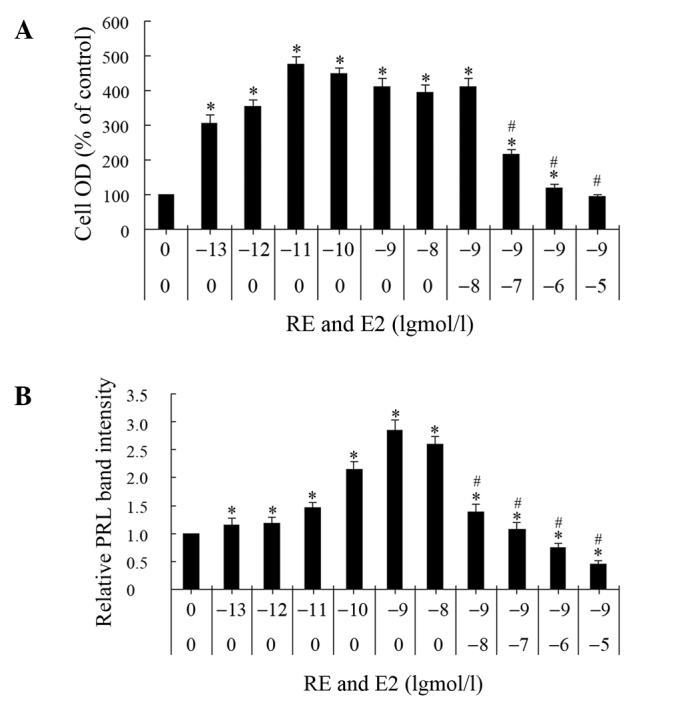
Differential effect of RE and E2 on PRL synthesis and cell proliferation. GH3 cells were incubated with either E2 alone or with RE. (A) Cell proliferation was assessed via an MTT assay. Data were calculated as a percentage of the control and expressed as the mean ± SD of three separate experiments. (B) Intracellular PRL was analyzed. The concentration of PRL in the cell lysate was determined by western blot analysis and equal loading was confirmed by determining the actin content. Each band was quantified using a densitometer. The value of the control was defined as 1.0 and from this value, the PLR values were calculated. The values represent the means ± SEM (bar) from the three independent methods. ^*^Indicates a significant difference from the control and ^#^indicates a significant difference from the 1 nM E2-treated group (P<0.01). OD, optical density; RE, resveratrol; PRL, prolactin; GH, growth hormone.

To determine the antiestrogenic effect of RE, GH3 cells were treated with E2 alone, or E2 and RE simultaneously, for three days and two parameters were analyzed, namely, cell proliferation and PRL synthesis (Fig. 5). The effect of the E2 concentration on cell proliferation was measured using an MTT assay. At the selected concentrations (0.1 pM-10 nM), E2 stimulated proliferation 2- to 4-fold with a maximum effect at 0.01 nM, whereas a higher concentration decreased cell growth. The cells maintained full levels of proliferation, comparable with those induced by E2 alone, when incubated with 1 nM E2 and 0.01 μM RE. However, 0.1 μM RE decreased the proliferation induced by 1 nM E2 and 10 μM RE inhibited the 1 nM E2-induced proliferation to levels comparable with those of the control group.

The effects of the E2 concentration on PRL synthesis were analyzed. At concentrations between 0.1 pM and 10 nM, E2 stimulated PRL synthesis. A concentration of 1 nM E2 resulted in a maximal response and the half maximal induction concentration (EC_50_) was ~0.01 nM. When GH3 cells were simultaneously treated with E2 and RE, with the latter at selected concentrations between 0.01 and 10 μM, RE inhibited the PRL synthesis that was induced by 1 nM E2 in a concentration-dependent manner; at a concentration of 0.01 μM, RE decreased the E2-induced intracellular PRL content to 50% of the level induced by E2 alone.

## Discussion

Estrogen plays a key role in the development and progression of pituitary prolactinoma (1). Selective estrogen receptor modulators (SERMs) exhibit antiestrogenic properties (8) and may be adopted in the treatment of prolactinoma. Numerous studies have identified that RE, a type of SERM, is able to induce growth inhibition and apoptosis in certain tumors including GH3 cells (14,15). Therefore, RE may be a potential therapeutic agent for prolactinoma. The aim of medical therapy for prolactinoma is to reduce the volume of the tumor and decrease the level of PRL to improve the endocrine symptoms.

The present study demonstrated the concentration- and treatment duration-dependent biphasic effect of RE on the proliferation of GH3 cells. After three days of treatment, RE stimulated cell proliferation at low concentrations (0.1–1 μM), whereas it inhibited cell proliferation at high concentrations (10–50 μM). The concentration of RE required for 50% inhibition of the GH3 cell proliferation, as compared with the control, was ~20 μM. When the treatment duration was reduced to 6 h, RE treatment inhibited proliferation in a concentration-dependent manner.

We previously observed the apoptosis in GH3 cells as a result of RE exposure (14). Chu *et al* (16) demonstrated that RE induced growth inhibition via cell cycle arrest and apoptosis in GH3 cells. However, the underlying molecular mechanisms were not clear. It was hypothesized that RE-induced cell death is tumor-specific and involves the cluster of differentiation 95 (CD95) or CD95-ligand system as the apoptotic trigger. In the present study, RE activated the caspase-8 and -3 pathway, which resulted in the cleavage of PARP. Therefore, RE-induced apoptosis in GH3 cells was shown to be caspase-dependent.

The results of the immunocytochemical experiments showed a decreased proportion of PRL^+^/GH^+^ cells and an increased proportion of GH^+^ cells following treatment with RE. Numerous studies have shown that PRL^+^/GH^+^ cells are capable of bipotential differentiation into PRL^+^ cells or GH^+^ cells when induced by specific growth factors (11,16,17). Lee *et al* (8) demonstrated that in GH3 cells, the percentage of PRL-immunopositive cells was increased by E2 and decreased by tamoxifen. Furthermore, E2 combined with epidermal growth factor and insulin, increases the percentage of PRL^+^/GH^+^ cells and stimulates the development of PRL^+^ cells (18). In physiological states of estrogen excess, such as pregnancy or the estrous phase of the estrous cycle, the percentage of lactotrophs increases in the pituitary. Prolactinoma growth occurs in ~23% of females harboring macroprolactinomas during pregnancy; therefore, estrogen is key in lactotroph proliferation and differentiation (1). In the present study, RE inhibited proliferation and, therefore, decreased the percentage of lactotrophs in GH3 cells, thus indicating that RE may affect the differentiation of GH3 cells.

E2 stimulated proliferation in GH3 cells at a low concentration (0.1 pM) and GH3 cells exhibited maximum growth with 0.01 nM E2 alone; however, greater concentrations decreased cell proliferation. When GH3 cells were simultaneously treated with 1 nM E2 and varying concentrations of RE (0.01–10 μM), 0.01 μM RE exhibited no effect on E2-induced proliferation. Conversely, at concentrations between 0.1 and 10 μM, RE inhibited the E2-induced proliferation. Kansra *et al* (19) demonstrated that E2-induced proliferation in GH3 cells may be mediated through ERα, which is capable of binding to RE with a 7,000-fold lower affinity than E2 (9). This may be the reason that, compared with the concentration required for stimulating proliferation by E2, only higher concentrations of RE exhibit the inhibitory effect on E2-induced proliferation.

RE is able to inhibit PRL gene expression, which may have contributed to the RE-induced reduction in the proportion of lactotrophs in GH3 cells. The present study demonstrated that E2 increased PRL production. E2, at a concentration of 1 nM resulted in a maximal PRL response and the EC_50_ was ~0.01 nM. Previous studies have demonstrated that the effects of E2 are mediated by ERα and ERβ (19,20).

E2 induced the proliferation of GH3 cells and increased PRL synthesis. GH3 cells showed maximum cell growth at 0.01 nM E2, whereas only half-maximal PRL production occurred at the same concentration. This indicates that the sensitivity of the PRL response to E2 was lower than that of the proliferation response. When these two responses were compared via inhibition of estrogen activity with increasing quantities of RE, the proliferation response was not as sensitive to antiestrogen as the PRL response was. The results indicate that a 0.01 μM concentration of RE was not able to inhibit 1 nM E2-induced proliferation, whereas an equal concentration of RE decreased PRL secretion to 50% of the 1 nM E2-induced, PRL secretion. Four explanations for these observations are: i) PRL and proliferation responses are mediated via different ERs; ii) RE and E2 possess different affinities for ERs; iii) ERs interact with a nuclear factor that is critical for replication at a greater affinity than its affinity for factors affecting PRL gene expression; iv) novel ER types exist (21). The inhibitory effect of RE on PRL synthesis may be useful in the treatment of prolactinoma, particularly with microprolactinoma where the predominant aim of therapy is to decrease the high level of PRL and improve endocrine symptoms.

In conclusion, RE exerts its antitumor effect on GH3 cells in two ways: Through the suppression of cell growth and by inducing a reduction in PRL expression. Furthermore, its antiestrogenic effects on cell proliferation and PRL synthesis indicate that RE may be effective in the chemoprevention and chemotherapy of pituitary prolactinoma.

## Figures and Tables

**Table I:** Proportion of each cell type in the GH3 cells, following treatment for three days at different concentrations of RE.

	Proportion (%) of each cell type		
	
Treatment	PRL^+^/GH^+^ cells	GH^+^ cells	PRL^−^/GH^−^ cells
Control	9.2±0.6	73.5±2.1	17.3±1.4
0.1 μM RE	9.1±0.4	73.9±1.6	17.0±0.6
0.5 μM RE	9.3±0.1	73.7±0.6	17.0±1.0
1.0 μM RE	9.2±0.4	73.4±2.1	17.4±0.7
10 μM RE	7.6±0.7^a^	75.2±1.6^a^	17.2±0.2
20 μM RE	7.4±0.1^a^	75.3±0.9^a^	17.3±0.7
50 μM RE	6.2±0.8^a^	75.7±1.8^a^	17.1±1.0

a Indicates a significant difference from the control (P<0.01).

The number of cells were determined following three days of treatment with RE and the proportions of each cell type were calculated as described in Materials and methods. GH, growth hormone; RE, resveratrol; PRL, prolactin. Data are expressed as mean ± SD.
